# Supplementary material for: Molecular Characterization of Subdomain Specification of Cochlear Duct Based on Foxg1 and Gata3
Source: Int J Mol Sci. 2024 Nov 26;25(23):12700. doi: 10.3390/ijms252312700 (PMC11640830; doi:10.3390/ijms252312700)
Supplement: Supplementary file 1 [file ijms-25-12700-s001.zip › ijms-3254008-supplementary.pdf]

Supplementary Information

Supplemental Figures

Figure S1

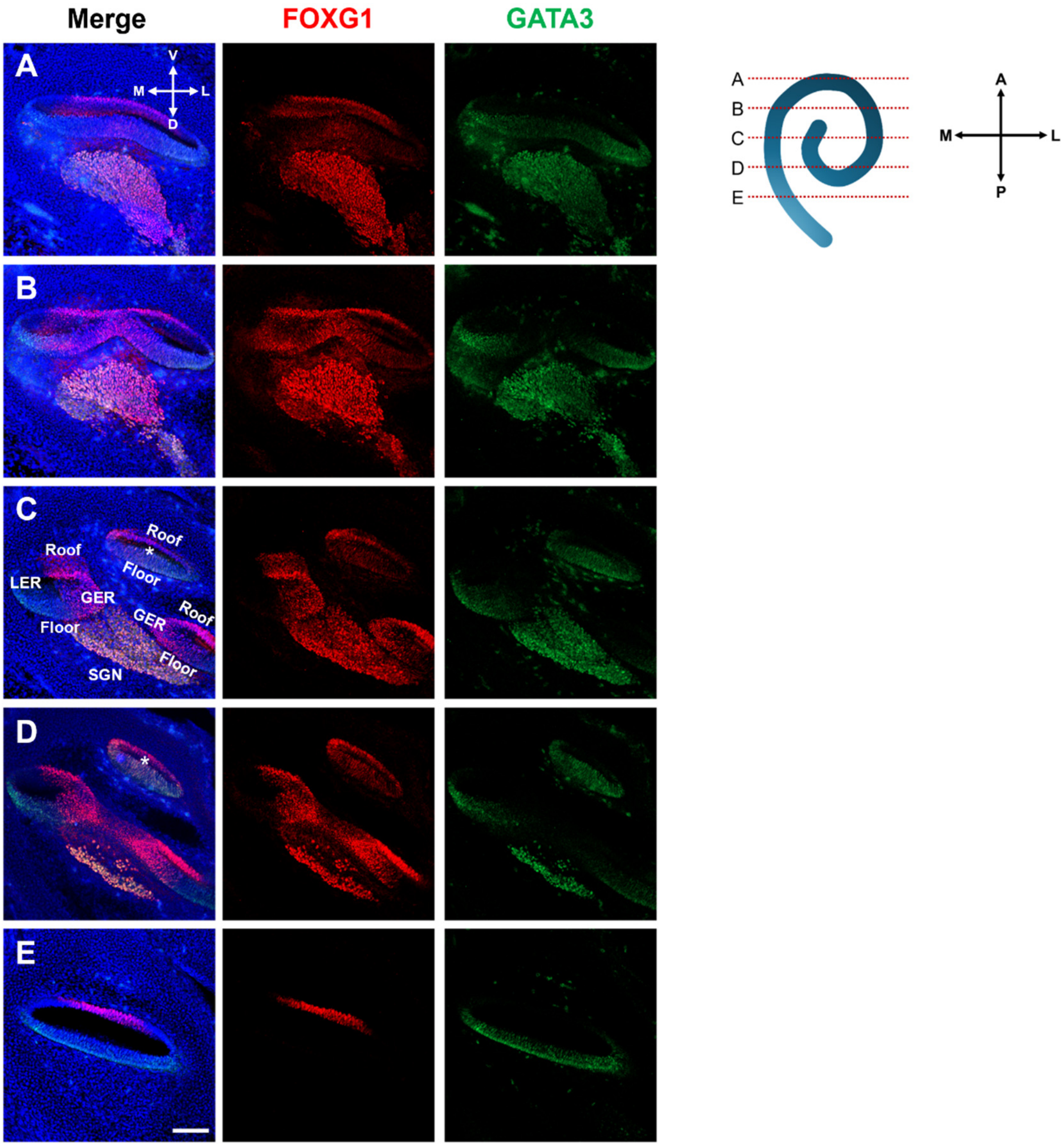

Figure S1. Foxg1, Gata3 expression patterns in serial sections of E13.5 cochlear duct. E13.5 serial section of the cochlear duct (A-E). Both Foxg1 and gata3 are expressed in the SVG near the cochlear duct, and in the cochlear duct, Foxg1 is expressed exclusively with gata3, but in the apical duct (asterisk in C,D), Foxg1 is expressed throughout. Scale bar=100μm. CD, cochlear duct; OV, otic vesicle; GER, greater epithelial ridge; LER, lesser epithelial ridge; SGN, spiral ganglion neurons.

Figure S2

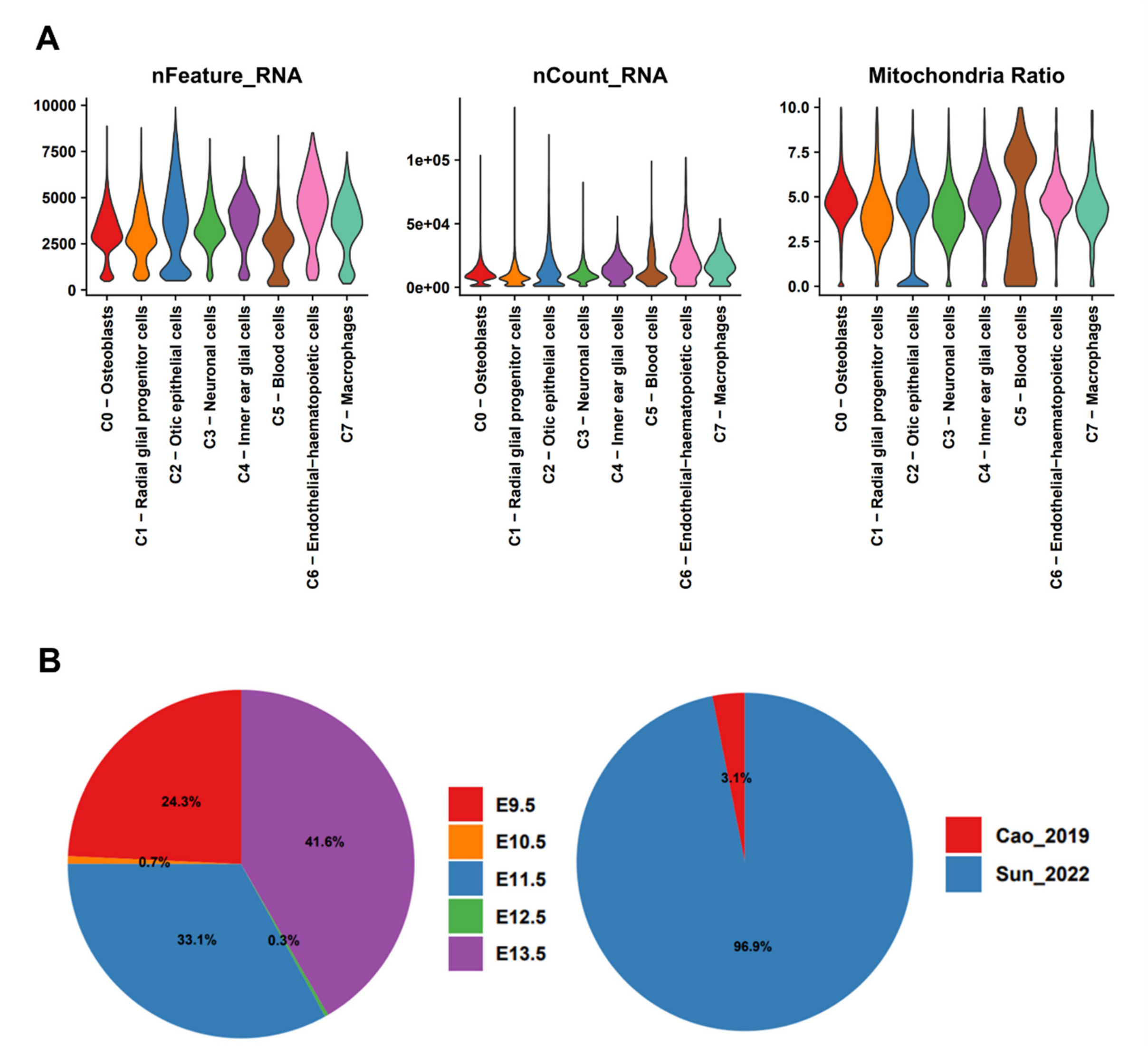

Figure S2. Quality control of single-cell data from the mouse inner ear. (A) Violin plots showing genes, read counts, and the percentage of mitochondrial genes per cell in each cluster. (B) Pie chart showing the proportion of cells by stage and reference.

Figure S3

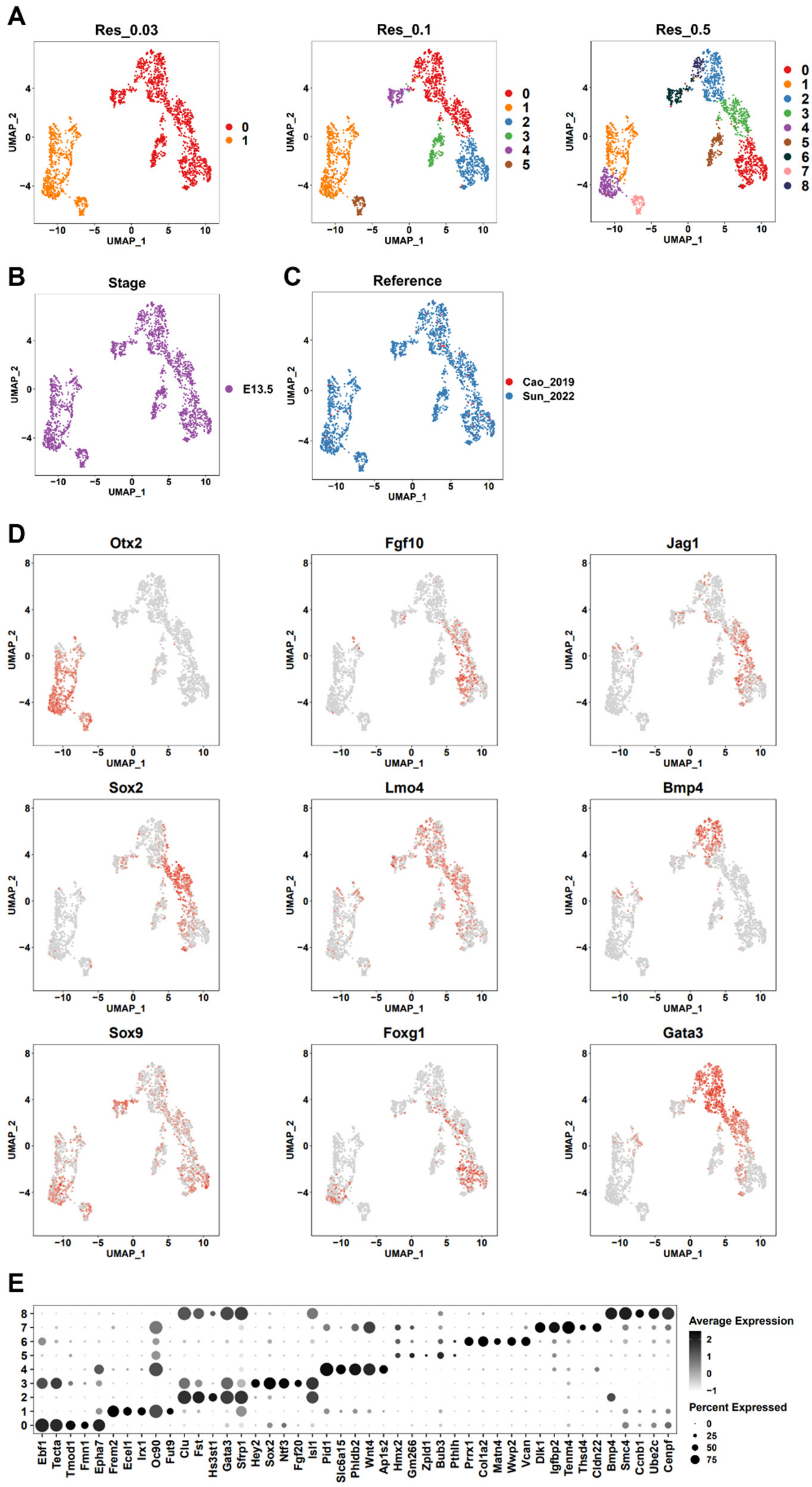
A
Res_0.03
Res_0.1
Res_0.5
UMAP_1
UMAP_2
B
Stage
E13.5
C
Reference
Cao_2019
Sun_2022
D
Otx2
Fgf10
Jag1
Sox2
Lmo4
Bmp4
Sox9
Foxg1
Gata3
E
Average Expression
Percent Expressed
Ebf1
Tecta
Tmod1
Fmn1
Epha7
Frem2
Ecel1
Irx1
Oc90
Fut9
Clu
Fst
Hs3st1
Gata3
Sfrp1
Hey2
Sox2
Ntf3
Fgf20
Isl1
Pid1
Slc6a15
Phldb2
Wnt4
Ap1s2
Hmx2
Gm266
Zpld1
Bub3
Pthlh
Prrx1
Col1a2
Matn4
Wwp2
Vcan
Dlk1
Igfbp2
Tenm4
Thsd4
Cldn22
Bmp4
Smc4
Ccnb1
Ube2c
Cenpf

Figure S3. Sub-clustering of E13.5 mouse cochlear duct cells. (A) UMAP plots showing clusters divided by resolution 0.03, 0.1, and 0.5. (B) – (C) UMAP plots for stage (B), reference (C). (D) UMAP plots showing the expression of marker genes, Foxg1, and Gata3. (E) Dot plots showing Top 5 marker gene expression and proportion in individual cell clusters. Color gradient representing average expression, and dot size indicating percent expressed cells.

Figure S4

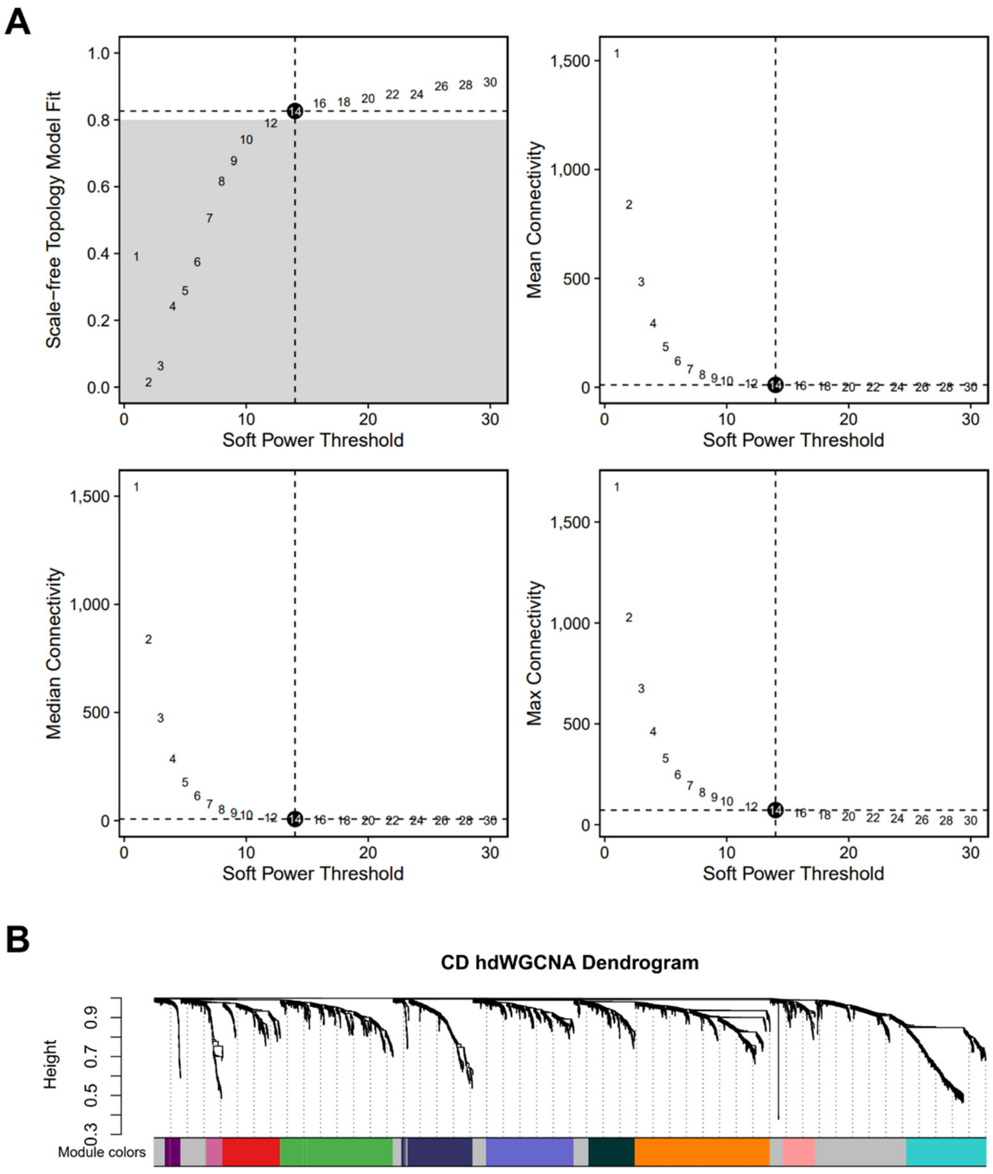

Figure S4. Pre-processing of hdWGCNA analysis. (A) Plot showing Scale-Free Topology Model Fit greater than or equal to 0.8 for the soft power threshold. (B) Dendrogram showing co-expression modules, with each leaf representing a gene and color indicating module assignment.

Figure S5

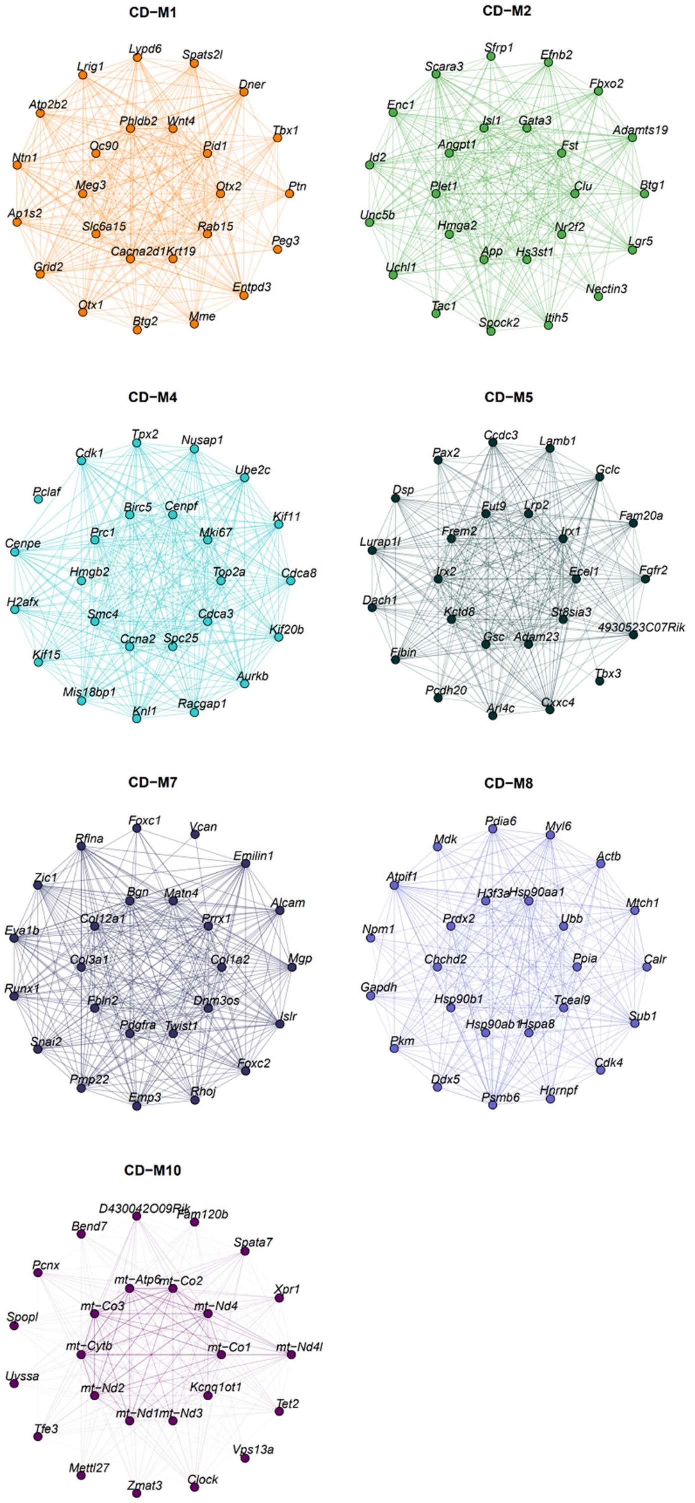

Figure S5. Hub gene network for each module identified by hdWGCNA. Gene network showing hub genes, with nodes colored by modules.

Figure S6

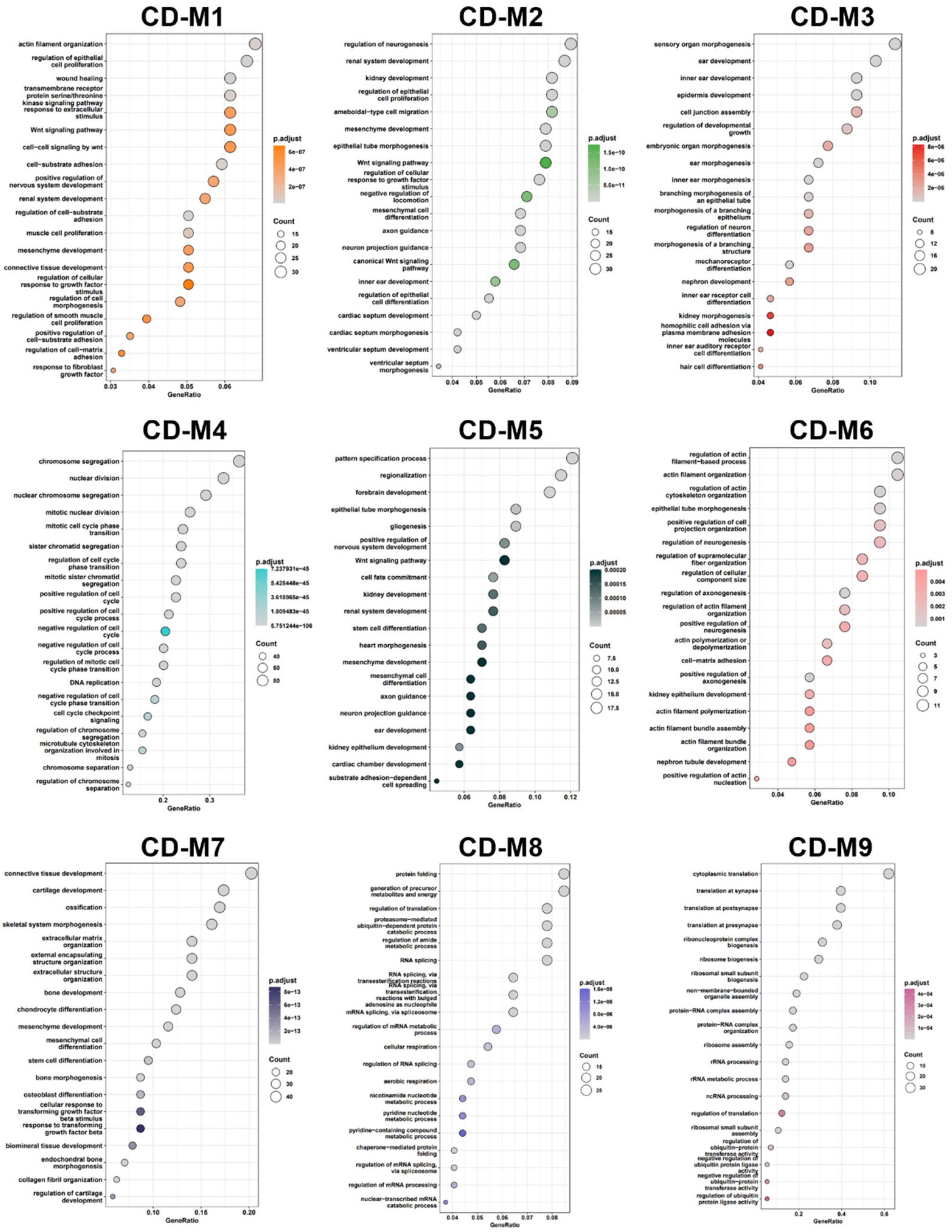

Figure S6. Gene Ontology analysis for each module identified by hdWGCNA. Dot plot showing Gene Ontology (GO) terms related to Biological Process for genes in each module. Dot size representing gene count, and color representing adjusted p-value.

Table S1. Hearing loss associated genes in modules

Gene	Disease	Module
Actg1	Baraitser-Winter syndrome	CD-M1
Actg1	NSHL	CD-M1
Edn3	Waardenburg syndrome	CD-M1
Ednrb	Waardenburg syndrome	CD-M1
Hsd17b4	Perrault syndrome	CD-M1
Myo6	NSHL	CD-M1
Clrn1	Usher syndrome	CD-M2
Col9a2	Stickler syndrome	CD-M2
Eps8	NSHL	CD-M2
Gata3	Hypoparathyroidism, SNHL, and renal dysplasia	CD-M2
Gjb2	NSHL	CD-M2
Gjb2	Skin phenotype and HL	CD-M2
Otogl	NSHL	CD-M2
Six1	Branchio-oto-renal syndrome	CD-M2
Ush1c	Usher syndrome	CD-M2
Ush1c	NSHL	CD-M2
Cdc14a	NSHL	CD-M3
Cdc14a	Hearing impairment and infertile male syndrome (HIIMS)	CD-M3
Chd7	CHARGE syndrome	CD-M3
Eya1	Branchio-oto-renal syndrome	CD-M3
Pcdh15	Usher syndrome	CD-M3
Pcdh15	NSHL	CD-M3
Slitrk6	Deafness and myopia	CD-M3
Tecta	NSHL	CD-M3
Diaph3	Auditory neuropathy spectrum disorder	CD-M4
Dnmt1	DNMT1 methylopathy	CD-M4
Tubb4b	Leber congenital amaurosis	CD-M4
Ccdc50	NSHL	CD-M5
Hgf	NSHL	CD-M5
Ror1	Hearing loss and auditory neuropathy	CD-M5
Tmtc2	NSHL	CD-M5
Espn	NSHL	CD-M6
Gsdme	NSHL	CD-M6
Otoa	NSHL	CD-M6
Col11a2	Otospondylomegaepiphyseal dysplasia	CD-M7
Col11a2	NSHL	CD-M7
Homer2	NSHL	CD-M7
Lars2	Perrault syndrome	CD-M7
Pou3f4	NSHL	CD-M7
Snai2	Waardenburg syndrome	CD-M7
Crym	NSHL	CD-M9

Figure S7

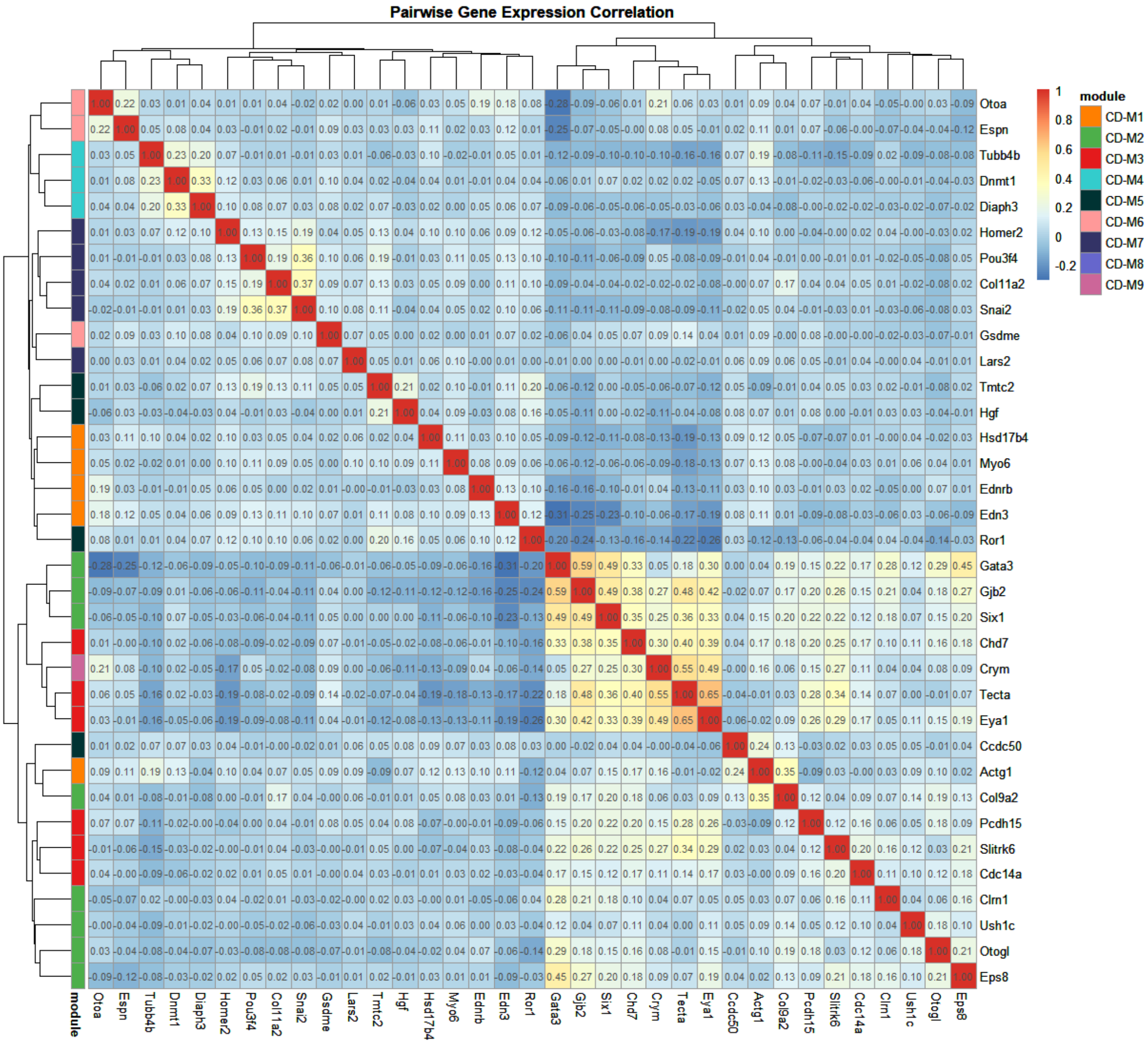

Figure S7. Correlation of Overlapping Genes Between Hearing Loss-Associated Genes and Modules. A pairwise correlation matrix of overlapping genes between those included in the hearing loss database(Clingen) and the genes within each module. In this analysis, hierarchical clustering was performed, and the color annotations on the left indicate modules.

Figure S8

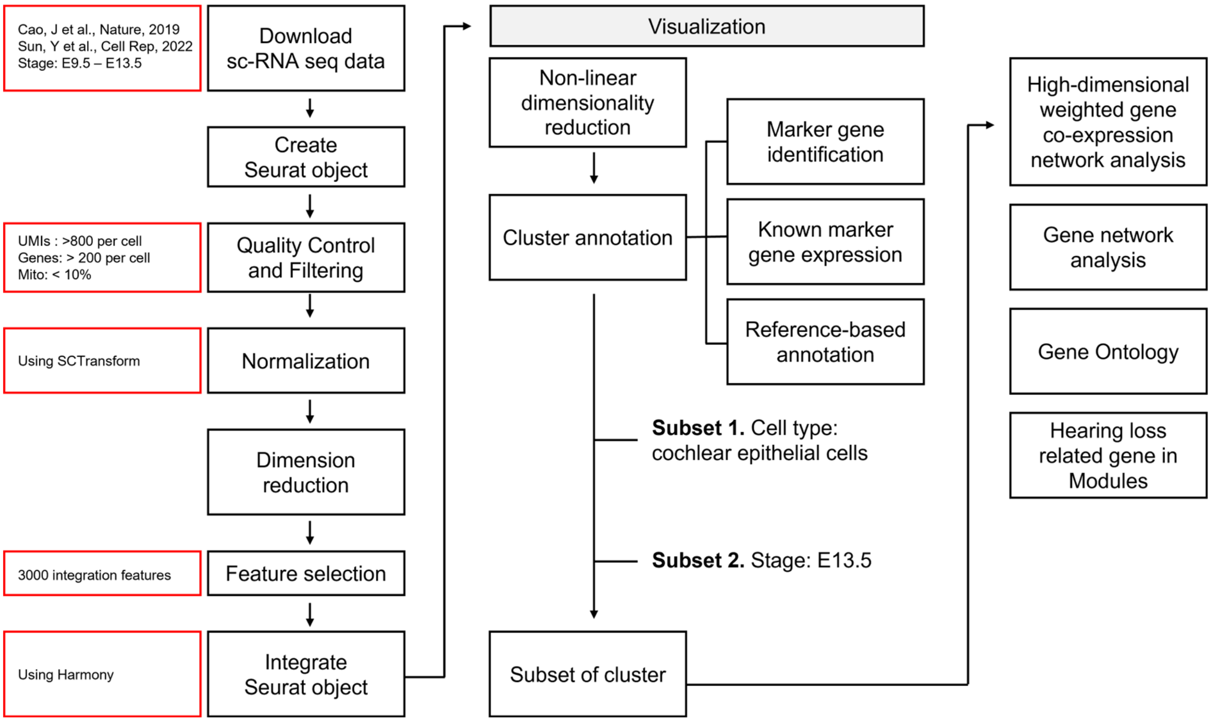

Figure S8. Schematic diagram of the single cell RNA seq analysis process. Overview of procedures used for scRNA-seq analysis. This analysis proceeded in the direction of the arrow. Each box represents a process, and the red boxes represent the process parameters and tools used (see methods).
